# Synthetic Biology Goes Cell-Free

**DOI:** 10.1186/s12915-019-0685-x

**Published:** 2019-08-08

**Authors:** Aidan Tinafar, Katariina Jaenes, Keith Pardee

**Affiliations:** Leslie Dan Faculty of Pharmacy, University of Toronto, 144 College St., Toronto, ON M5S 3M2 Canada

## Abstract

Cell-free systems (CFS) have recently evolved into key platforms for synthetic biology applications. Many synthetic biology tools have traditionally relied on cell-based systems, and while their adoption has shown great progress, the constraints inherent to the use of cellular hosts have limited their reach and scope. Cell-free systems, which can be thought of as programmable liquids, have removed many of these complexities and have brought about exciting opportunities for rational design and manipulation of biological systems. Here we review how these simple and accessible enzymatic systems are poised to accelerate the rate of advancement in synthetic biology and, more broadly, biotechnology.

## Moving towards a new bioengineering platform

Since its emergence, the field of synthetic biology has given rise to the development of many technologies that are implemented using the whole cell [1]. These have included biosensors capable of detecting broad ranges of analytes [2–5], systems that can count [6] or perform complex logic [7–10], engines for the bioproduction of valuable commodities [11–14], gene-circuit-driven chassis for regenerative medicine [15, 16], and engineered CAR-T cells [17]. Such technologies are on track to transform many aspects of modern life, yet their requirement for a cellular host has limited their reach and scope. For example, concerns over biosafety have restricted the use of engineered cells, and the systems they host, largely to laboratory settings. The self-replicability of cell-based systems carries the risk of “escape” or contamination that could impact human health, food security, and the environment. While the development of safeguards to prevent these types of events is an active area of research [18, 19], failure-free implementation of such systems is not a trivial task.

Another substantial limitation of cell-based synthetic biology is the requirement for laborious genetic encoding of its design features into a living cell, which can limit its functionality and significantly slow down design–build–test cycles. In cell-based systems, genetic instructions often need to be assembled into a vector, imported into the cell, and maintained by using a selectable marker or by genomic integration. Only then can the instructions be evaluated. Furthermore, designs must be iteratively tested to minimize cross-talk with endogenous molecular programs while balancing between the metabolic burden on the cellular host and the desired outcome.

Cell-free systems offer a means to circumvent many of these limitations. They were originally conceived as tools to facilitate in vitro protein synthesis and consist of molecular machinery extracted from cells. They typically contain enzymes necessary for transcription and translation, and accordingly are able to perform the fundamental processes of the central dogma (DNA➔RNA➔protein) independent of a cell. These systems can be derived from eukaryotes (e.g., vertebrates, plants, insects, fungi) [20–27] or prokaryotes (e.g., *Escherichia coli*, *Vibrio natriegens*, *Bacillus subtilis*) [28–43] and may be prepared as either purified components [36, 44] or semi-processed cellular extracts [38]. CFS can be made sterile via simple filtration, which provides for a biosafe format for use outside of the lab.

The open nature of CFS means that there is no physical barrier (e.g., a cell wall) to programming and modification. CFS can be augmented with proteins or small molecules that improve the performance of synthetic gene networks [45, 46] or the productivity of reactions [39, 47]. More importantly, genetically encoded instructions can be added directly to CFS at desired concentrations and stoichiometries using linear or circular formats. This means that conceptual designs can go from computational instructions to chemical synthesis and amplification (e.g., through PCR) to CFS without the need for selective markers or cell-based cloning steps. Such simplicity allows for rapid prototyping of molecular tools.

Importantly, CFS can be freeze-dried, enabling room temperature storage and distribution [46, 48]. Freeze-dried cell-free (FD-CF) systems can then be activated at the time of need simply by adding water [46]. This feature has been used to deploy biosafe, genetically encoded tools outside of the laboratory as diagnostics and as platforms for biomanufacturing [49, 50], as well as their deployment in altogether new contexts, such as global health and education.

**Fig. 1:**
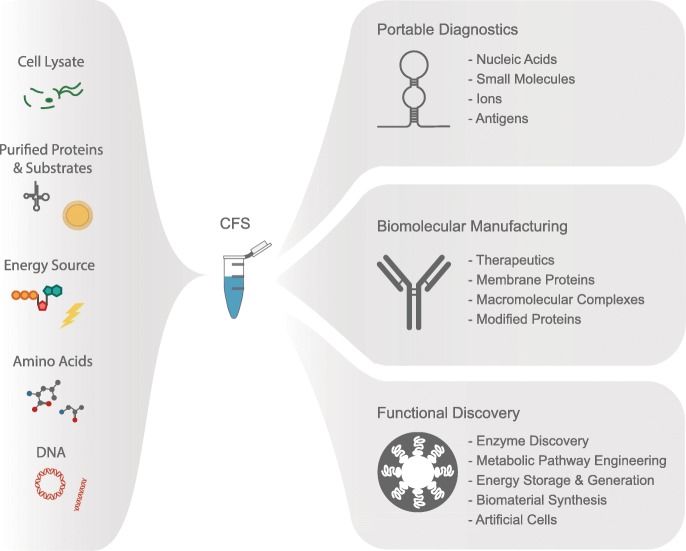
Cell-free protein expression systems and their applications. Capitalizing on their open nature, CFS can be rationally assembled to include cell lysates, purified proteins, energy sources (e.g., ATP), amino acids, other substrates (such as modified tRNAs and membrane mimics) and RNA or DNA (circular or linear). CFS can be applied in portable diagnostic devices [46, 50] and also hold great potential for biomolecular manufacturing [49, 51]. Additionally, CFS can enable discovery of novel enzymes (e.g., through directed evolution) [52]

Below we will discuss how CFS are enabling new technologies and accelerating the coming revolution in bioengineering, highlighting some of the most active areas of research in the cell-free community (Fig. 1).

## Development of sensors

Molecular recognition underlies almost every biological process, including the nucleic acid base pairing that imparts specific syntax to the central dogma. Scientists and engineers have long worked to usher these processes into cell-free in vitro environments to understand and exploit their underlying molecular mechanisms for purposes such as diagnostics and detection of molecules. One of the fruits from such efforts is the polymerase chain reaction (PCR), which is now an indispensable tool utilized in most molecular biology laboratories, including those for clinical diagnostics. There is currently a growing need for de-centralized, portable diagnostics that can be rapidly deployed in the field, for instance during infectious disease outbreaks or for agricultural purposes. However, sensing technologies such as PCR and others have largely remained confined to laboratories in large urban centers due to their requirement for specialized equipment and personnel.

The biosafe and stable nature of FD-CF systems offers an alternative molecular venue to address the unmet need for distributed and low-cost sensing. Here, the transcription and translation properties of CFS can be used to host gene circuit-based sensors that can detect small molecules and nucleic acids with exquisite sensitivity and specificity. Many of the biosensors and circuits that have been developed for cell-based applications can be operated in the cell-free environment. These include, among others, many classic switches (e.g., TetO- and LacI-based systems), logic gates, negative feedback loops, transcriptional cascades [37, 41, 53–56] and ring oscillators [57]. This cross-compatibility between CFS and cell-based systems has also been exploited for rapid prototyping of regulatory elements that can be brought back to the cell-based environment.

FD-CF systems do not require a temperature-controlled environment and cold-chain logistics intrinsic to many other diagnostic approaches, as they remain active for at least a year without refrigeration, enabling room temperature storage and distribution [46]. This, however, does not circumvent the challenges arising from handling these molecular tools in liquid phase—for instance upon their resuspension outside of the laboratory environment. Inspired by systems like pH paper and lateral-flow diagnostics, we embedded FD-CF reactions into porous materials (e.g., paper), demonstrating that low-volume reactions (1–2 μL) could readily be achieved within this medium. Such paper-based cell-free systems enabled the deployment of poised synthetic gene networks outside of the laboratory in a contained and biosafe format for the first time [46].

With this new ruggedized paper-based format, simple sensing such as anhydrotetracycline (ATc)-inducible expression of GFP and mCherry was established [46]. However, to demonstrate the real-world potential for this system, a sensing platform that could be rationally designed to detect a wide range of practical analytes was needed. This was realized with the introduction of toehold switches [58], a new class of riboregulators, into FD-CF reactions. The use of toehold switches, which can be designed to recognize virtually any sequence of interest, was first demonstrated in paper-based FD-CF reactions for the detection of genes responsible for antibiotic resistance and strain-specific detection of the Ebola virus [46]. While the demonstration of this sensing capacity in a portable format was exciting, the system lacked the sensitivity necessary to detect RNA levels generally present in patient samples.

This sensitivity challenge was addressed by placing an isothermal amplification step (e.g., NASBA) in the workflow upstream of the cell-free reaction. This improved the threshold of detection by orders of magnitude (10^6^). Since isothermal amplification is a primer-directed process, combination with toehold-based sensing results in two sequence-specific checkpoints. An opportunity to test out the improved system presented itself in early 2016 when the outbreak of the mosquito-borne Zika virus was reported in Brazil. With the improved embodiment, FD-CF toehold sensors could detect all global strains of the Zika virus at clinically relevant concentrations (down to 2.8 femtomolar) from viremic plasma [50]. Moreover, powered by the first CRISPR-based system in an in vitro diagnostic system, viral genotypes could be distinguished with single base pair resolution (e.g., American vs African Zika strains). Most recently the Collins group extended these concepts in a *tour de force* effort that demonstrated quantitative detection of ten gut bacterial species from patient samples [59]. This work demonstrated detection at clinically relevant concentrations with sensing performance that mapped well with parallel measurements done with RT-qPCR. It also showcased the ability to detect a toxin-related sequence for the diagnosis of *Clostridium difficile* infections.

**Fig. 2:**
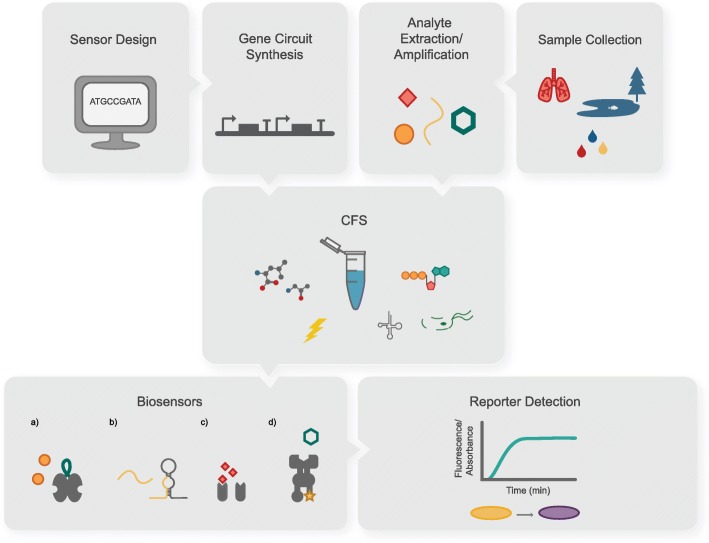
Overview of the use of biosensors in CFS. The general workflow usually involves in silico design of gene circuits encoding biosensors and reporter proteins, followed by chemical synthesis of such circuits. Meanwhile, patient or environmental samples are collected, target analytes are extracted, and, in some cases, amplified. The gene circuits and target analytes are then added to CFS. Examples of biosensors in CFS have included **a**) mercury (II) detection using the MerR repressor[45], **b**) viral and bacterial nucleic acid sensing using toehold switch-based sensors [46, 50, 59], **c**) identification of *P. aeruginosa* infection by its quorum sensing molecule, 3-oxo-C12-HSL, using the LasRV sensor [61] and **d**) recognition of an endocrine-disrupting compound by utilizing an allosterically activated fusion protein containing the ligand binding domain of a human estrogen receptor [62, 63]. Reporters (e.g., colorimetric or fluorescent) can then produced, contingent upon analyte detection, enabling clinical diagnosis (e.g., using standard spectrophotometers)

Following the initial work outlining the potential for the FD-CF format, a body of work ensued demonstrating many biosensing applications and improvements on FD-CF preparations. In one of the earliest examples, Duyen et al. developed a sensor for the detection of antibiotic contamination based on protein synthesis inhibition caused by some antibiotics [60]. The Freemont group applied their expertise in CFS to develop sensors for the detection of *Pseudomonas aeruginosa* in cystic fibrosis patient samples [61], demonstrating that the quorum-sensing molecule from *P. aeruginosa* (3-oxo-C12-HSL) could be detected down to low nanomolar concentrations. Another novel approach used CFS to express engineered protein fusions containing nuclear receptor ligand binding domains for the detection of endocrine-disrupting compounds [62, 63]. This work showcased sensitivity in the nanomolar range, and, interestingly, demonstrated that CFS could operate in the presence of contaminants in environmental and clinical samples. In another example, detection of mercury contamination using the mercury(II)-responsive transcriptional repressor MerR was accomplished [45] (Fig. 2).

## Manufacturing of therapeutics

Another active area in CFS research is the biomanufacturing of therapeutics and other protein-based reagents. Natural biological systems have evolved a remarkable capacity to synthesize a variety of molecules ranging from metabolites to biopolymers. Cell-free protein expression systems allow the incorporation of such reactions into a highly controlled process that allows production of molecules as needed and in the field. Our primary focus here will be on a subset of biopolymers, namely therapeutic proteins. The ongoing work in this field rests on decades of research that have led to the productive and practical systems currently available [28, 29, 36–38, 40]. Recent advances in high-throughput preparation techniques [40, 45] and in the development of systems that can use more economical energy sources [64, 65] have made CFS highly accessible. Meanwhile, significant strides are being made towards resolving various protein folding issues and shortcomings in post-translational modifications [66] associated with traditional CFS. Recent advances have showcased the potential for scaling up cell-free reactions, with some having demonstrated reaction volumes reaching 100 liters [67, 68] to 1000 liters [69]. Cell-free expression has been used as a platform for the production of a wide range of potential therapeutics, some of which have been summarized in Table 1. A number of these products have been validated in animal models [49, 76].

**Table 1:** Examples of potential therapeutics expressed in CFS to date

Therapeutic proteins	Granulocyte macrophage colony-stimulating factor (GM-CSF) [68, 70]
	Erythropoietin [70–72]
	Cytotoxic protein onconase [73]
	Antibodies [51, 74, 75] and antibody fragments [49, 76–79]
	Bispecific antibodies [80]
	Antibody-drug conjugates [49, 81]
	Tissue-type plasminogen activator [82–85]
Vaccine antigens	Picornaviral capsid intermediate structures [86]
	Trimeric influenza hemagglutinin head [87] and stem [88] proteins
	Trivalent vaccine based on Hc fragments of botulinum toxins A, B, and E [89]
	Anthrax protective antigen and diphtheria toxoid [49]
Virus-like particles	A B-cell lymphoma vaccine [90]
	Anti-hepatitis B VLPs [91]
	A virus-like nanoparticle scaffold for vaccines and drug delivery [92]
Antimicrobials	Antimicrobial peptides [49, 93]
	Small molecules such as violacein [49, 56]

Two primary modes of CFS have been pursued. The first, used by commercial efforts such as Sutro [94], focuses on large, centralized production. This approach leverages the advantages of synthesis outside of the cell for biomanufacturing. For these applications, CFS not only allow for rapid production, but also significantly speed up the drug development process [95]. Remarkably, Sutro has reportedly increased their cell-free production to an incredible 1000 liters [69], showcasing the scalability of centralized cell-free production. The second mode uses FD-CF systems to de-centralize biomanufacturing capacity for small-batch production of therapeutics, with applications in global health and emergency response [49, 73, 96, 97]. Using this mode of production, we have recently demonstrated the proof-of-concept capacity to manufacture over 50 therapeutics and lab reagents, including proteins (e.g., vaccines, antibodies, and antimicrobial peptides) and small molecules [49], with applications outside of the laboratory setting.

Cell-free biomanufacturing is particularly well-suited for vaccine production due to its potential for rapid scale-up in response to public health emergencies. Successful cell-free expression of a number of recombinant vaccines (e.g., botulinum, diphtheria, anthrax) has been demonstrated [49, 86–90, 98], with some having been validated in animal models, such as mice [49, 90]. Considering the low dose requirements (microgram range) for many of these therapeutics, commercialization of CFS-derived vaccines will likely see rapid growth in the coming years. Production of antibodies has also been an area of focus for the cell-free community [20, 49, 51, 74–80, 99, 100]. Due to their compact size and relatively high expression levels in CFS, single-domain antibodies have garnered particular attention and seem strategically well-placed to serve the emerging needs in personalized medicine, i.e., for therapeutics and diagnostics.

Antibiotic resistance has been recognized as a major threat to global health, resulting in approximately two million illnesses and 23,000 deaths in the US alone every year [101]. Accordingly, cell-free production of antimicrobial compounds, including antimicrobial peptides and small molecule drugs, has become the focus of some groups [49, 93]. A number of labs have also demonstrated the power of CFS to express phages [56, 102–104]. The upward trend in the reported antibiotic resistance cases has led to a resurgence in viewing phage therapy as a potentially viable alternative to current antibiotic regimens [101, 105]. The use of phages has also been evaluated as an effective treatment strategy for a number of plant diseases, with some phages now being commercially available for mass consumption [106]. CFS-based production of these non-traditional antimicrobials could play a significant role in battling the antibiotic resistance crisis and could also help improve food security around the globe.

Below, we will highlight some of the areas in which CFS have shown great potential for enhancing current methods of therapeutics development and manufacturing. These advances are rapidly transforming CFS into an integral part of the manufacturing ecosystem.

### Membrane proteins

While approximately 70% of all drugs act on membrane proteins[107], working with these proteins is notoriously difficult due to their enrichment in hydrophobic surfaces. Cell-based expression of membrane proteins is often fraught with challenges, such as toxicity caused by their membrane incorporation or their incompatibility with the host’s physiology [108]. Recently, cell-free approaches have been used to tackle this challenging category of proteins, the coding sequences of which comprise 20–30% of all known genes [107]. When compared to current cell-based methods, CFS can be a powerful tool in the production of soluble active membrane proteins [109]. The ability to integrate steps that can tackle the challenging aspects of membrane protein synthesis is particularly valuable. For instance, previous efforts in cell-based systems have demonstrated that membrane mimics can be successfully used to synthesize and stabilize a wide range of membrane proteins such as G-protein-coupled receptors [110, 111], the epidermal growth factor receptor [71], hepatitis C virus membrane proteins [112], and an ATP synthase [109, 113]. These mimics include surfactants, liposomes, and nanodiscs [114–116] and can be added directly to CFS co-translationally or post-translationally. There is also evidence suggesting that functioning single-span membrane proteins can be synthesized simply in the presence of an oil–water interface (e.g., through the use of emulsions) [117].

### Macromolecular production

**Fig. 3:**
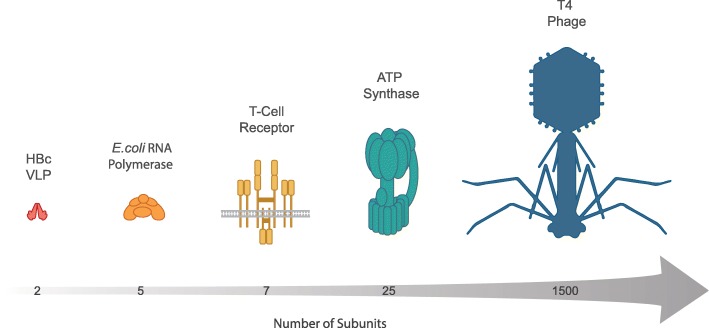
Multi-subunit protein complex synthesis in CFS. Various groups have demonstrated the production of increasingly intricate protein complexes. These have included the hepatitis B core antigen (HBc) VLP (2 subunits) [91], the *E. coli* RNA polymerase (5 subunits) [118], the human T-cell receptor (7 subunits) [119], an ATP synthase (25 subunits) [113], and the T4 phage (1500 subunits) [102–104]

Molecular research has highlighted the importance of protein–protein interactions and the resulting complexes that these interactions can generate. Whether it is for the biophysical study of these complexes or as vehicles for new therapeutic delivery (e.g., virus-like scaffolds for vaccines), there is a growing need for developing robust tools aimed at synthesis of such complexes. As in the case of membrane proteins, CFS have also demonstrated higher yields, compared to in vivo strategies, in the production of macromolecular assemblies such as virus-like particles (VLPs) [109]. Groundbreaking work by the Swartz group, demonstrating the cell-free expression of hepatitis B core antigen VLP (2 subunits) [91] in an *E. coli*-based cell-free system, opened the door to other researchers expressing a variety of macromolecular assemblies including the *E. coli* RNA polymerase (5 subunits) [118] and an ATP synthase (25 subunits) [113]. Earlier work with reticulocyte lysate had also demonstrated cell-free expression of the human T-cell receptor (7 subunits) [119]. Remarkably, a number of bacteriophages have now also been successfully expressed in CFS, including the T4 phage, which structurally contains 1500 proteins from 50 genes [56, 102–104] (Fig. 3).

Non-identical subunits of a protein complex are often referred to as hetero subunits. In some instances, such hetero subunits require co-translation to yield active complexes [120]. Thus, the ability of CFS to concurrently translate multiple mRNAs facilitates the production of active complexes composed of a number of different subunits [121]. Some CFS such as *E. coli*-based preparations are generally not capable of producing proteins that contain disulfide bonds, which are critical to numerous pharmaceutically relevant proteins (e.g., antibodies and many cytokines) [121]. However, recent efforts have augmented these systems to enable the production of complex proteins requiring multiple disulfide bonds [85, 99, 122], expanding the range of therapies that can be made in CFS.

### Modification of proteins and codon tables

Effectiveness of many protein-based therapeutics hinges upon precise control over natural or non-natural modification of their peptide sequences. One of the most compelling uses of such modifications is in the development of antibody−drug conjugates (ADCs), which are quickly gaining favor as a new class of therapeutics against cancer. Classic conjugation techniques result in a heterogeneous mixture of labeled antibodies due to their reliance on arbitrary conjugation to multiple amino acid side chains. Recent studies, however, suggest that pharmacologic properties of ADCs could be improved through site-specific conjugation. Non-natural amino acids provide an efficient avenue for such site-specific conjugation [123]. To date, co-translational incorporation of over 100 different non-natural amino acids has been demonstrated in vivo [124], allowing for a wide range of modifications [125–129]. Many of these modifications have been demonstrated in the cell-free context for a variety of applications, including orientation-controlled immobilization [92, 98] and site-specific functionalization (e.g., phosphorylation [130], PEGylation [131], or drug conjugation [81]) [132–134].

CFS platforms circumvent some of the cell-based toxicity and permeability limitations and offer greater control and versatility in making protein modifications [109, 135]. Incorporation of non-natural amino acids in cell-based approaches has typically relied on repurposing stop codons to minimize the negative impacts of recoding on cell-viability [109]. In a cell-free system, however, the entire codon table can in theory be reprogrammed, allowing not only for the incorporation of non-natural amino acids, but also for the creation of entirely novel codon tables.

Taken to its extreme, the latter could help with the protection of intellectual property. DNA sequences could be obfuscated such that they are rendered non-functional outside of their specialized cell-free context. This obfuscated code would make proprietary designs difficult to copy. Codon obfuscation could also pose serious challenges for the detection of DNA sequences that may be employed by malevolent entities. For example, DNA synthesis companies would have a much more difficult time screening against DNA sequences that could be used for nefarious activities (e.g., bioterrorism). Recent work has shown that the size of the codon table can also be expanded by augmenting the four-letter genetic alphabet with unnatural base pairs [136, 137]. Thus, proteins made in CFS could—at least in theory—hold an unlimited number of non-natural amino acids.

**Fig. 4:**
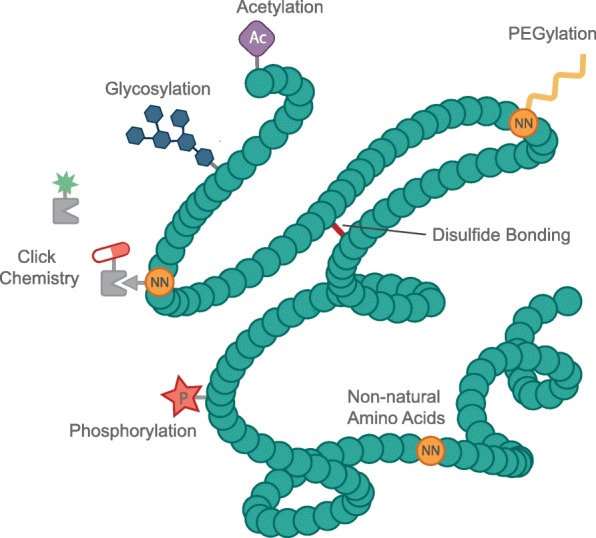
Protein modifications in CFS. Possible protein modifications include but are not limited to glycosylation, disulfide-bond formation, acetylation [140], phosphorylation [141], and PEGylation [131] (which may be accomplished through the use of non-natural amino acids). Non-natural amino acids can also be used for the conjugation of a wide range of compounds such as drugs (e.g., through click chemistry) [81] or fluorescent molecules [142]. Figure adapted from Pagel et al*.* [143]

CFS can also be employed for making naturally occurring modifications to proteins. An example of these is the grafting of sugars (i.e., glycans) referred to as glycosylation. Successful production of many therapeutics is often contingent upon highly efficient glycosylation, as lack of proper glycosylation can reduce the efficacy and circulation half-life of many therapeutic proteins [138]. Some CFS (e.g., insect, Chinese hamster ovary, and human K562 extract-based systems) are inherently capable of glycosylation. However, their repertoire of glycan structures tends to be limited to those naturally synthesized by their lysates’ source cell type. Additionally, glycosylation in these systems often requires recapitulation of the source cell’s protein trafficking mechanisms [109]. Thus, creation of synthetic glycosylation pathways in CFS has become an area of focus in recent years [135, 139]. Success in this domain will likely serve as a key catalyst in bringing cell-free-produced vaccines and other therapeutics to the masses. Figure 4 outlines some of the possible protein modifications in CFS.

### Directed evolution

Directed evolution is a powerful tool for aptamer and protein engineering that uses iterative rounds of mutagenesis and selection to modify or tune specific bimolecular properties (e.g., an enzyme’s substrate activity). Utility of aptamers or proteins, in a given context, with respect to their corresponding nucleotide sequences is often described as a fitness landscape. Directed evolution provides a massively parallel method for searching through a fitness landscape to find optimal variants and their corresponding genotypes [144]. This generally requires one-to-one mapping of phenotype to genotype. Although cells have a built-in capacity for such mapping due to their compartmentalized nature, using cells to conduct directed evolution can impose limits on the size of candidate libraries screened, and restricts the type of solvents, buffers, and temperatures that can be sampled [145]. As a result, cell-free directed evolution platforms have gained favor [145], starting with the first truly cell-free systems published in the late 90s [146, 147]. More recently, connecting phenotype to genotype has been accomplished through artificial compartmentalization (e.g., using emulsion, microbeads, and liposomes) [145, 148–151]. Applications have included the design and optimization of Fab antibody fragments [77, 152], membrane proteins [151], and, as we will discuss below, enzyme discovery [52].

## Platform for discovery

Engineered transcription and translation systems can also greatly catalyze research in the laboratory. As previously mentioned, the absence of a cell wall means that candidate genes can be readily screened for function. It also means that substrates, including those difficult to use in the cellular context, can easily be brought into contact with enzyme libraries to screen for novel reactions. Below we look at some of the recent work using CFS as a platform for discovery.

### Biosynthetic pathways

From the early days of synthetic biology, it was clear that there was great potential for synergy with the field of chemical synthesis. Metabolic pathways responsible for the synthesis of valuable compounds (e.g., drugs, scents, and flavors) were thus moved out of organisms that did not easily lend themselves to production and into heterologous hosts, such as yeast. This microorganism-based approach has been incredibly successful and has led to the assembly of genes from disparate sources to create engineered pathways. Enzyme-based catalysis has the advantage of allowing for stereo-selectivity in aqueous, low-energy reactions (e.g., green chemistry) [153]. By leveraging naturally occurring pathways, it has been possible to generate tremendous chemical diversity, as seen in isoprenoids, from simple precursors [154]. An exemplar of this approach is the synthesis of amorpha-4,11-diene and artemisinic acid, which are precursors to the anti-malarial compound artemisinin [154–157]. This process has been repeated for other pharmaceutical pathways, enabling the production of opioids [158, 159] and taxol [160], as well as for the generation of molecules for the energy industry and the agriculture sector [13, 161].

While microorganisms are currently a mainstay for biomanufacturing of commodities, their use for these purposes is nontrivial. For example, assembly, fine-tuning, and host strain integration of the industrialized pathway for the bioproduction of artemisinic acid is estimated to have taken over 150 person-years [162]. Another challenge to microbial bioproduction is that a significant portion of inputs are lost to general cellular metabolism and growth, reducing efficiency of the overall process [67, 134]. Cell-free synthetic biology alleviates some of these challenges. For instance, enzyme discovery—the identification of enzymes that can be used for biosynthetic purposes—via CFS has proven to be effective. Enzymes and their homologs can be rapidly screened for performance without the cumbersome steps required for cell-based screening (e.g., plasmid assembly and transformation). This approach can be extended to simple prototyping of pathways or the automated multiplexed shuffling of complex pathway components. Unlike with cell-based prototyping, the cell-free environment allows for the use of enzymes encoded as linear constructs (DNA or RNA). Substrate preference can also be evaluated without the need for enzyme purification.

In many cases, enzymes and pathways discovered in CFS will be brought back into cells for scale-up [163]. However, there is a growing case for using CFS directly as the production medium. Commercial ventures (e.g., Sutro, Greenlight) have already demonstrated that CFS can provide economic advantages for the production of protein and RNA products [69]. Thus, it would be reasonable to think that a similar approach could provide a viable source of high-value small molecules. Such systems have the advantage of enabling bioproduction without metabolic inefficiencies, toxicity limitations, complex extraction steps, or the need for integration into a host strain [67, 134, 164]. Combined with the capacity for efficient prototyping, these systems are generating significant enthusiasm. The field is now beginning to focus on more complex pathways (more than eight enzymes) and larger reaction volumes (> 100 L) [67].

Single enzyme reactions are highly simplified cell-free systems that have been used for decades at scale for washing (e.g., dish and laundry detergents) and for processing food, wood pulp, and fuel [165]. Once fully operationalized, more complex cell-free enzymatic pathways could revolutionize the chemical industry and enable greater accessibility to bioproduction. Earlier attempts at engineering such pathways outside of a cell were generally made by using purified components. These pathways have included those designed for the production of amorpha-4,11-diene [166], isoprene [167], fatty acids [168], and nucleotides [169]. Recent work has showcased the use of 27 purified enzymes that can work together to convert glucose into terpenes such as limonene, pinene, and sabinene [170]. Here, production can operate continuously for 5 days with a single addition of glucose, with glucose conversion of greater than 95%, to generate high product concentrations (> 15 g/L) that are well above levels toxic to microbes. While exciting, expression and purification of each individual component for such an approach is quite laborious.

Transitioning these metabolic pathways into CFS, where expression of enzyme-encoding sequences could lead to the self-assembly of pathways, would be incredibly enabling. To date, a number of reports have validated this approach. Three- and six-enzyme pathways have recently been generated de novo from DNA inputs in CFS to produce N-acetylglucosamine and a peptidoglycan precursor, respectively [171, 172]. A five-enzyme pathway that transforms tryptophan into a bioactive pigment called violacein has also been demonstrated [49, 56]. Additionally, a combinatorial strategy has recently been used to build a 17-step enzyme pathway for n-butanol [173]. It is intriguing to envision how this approach could influence the synthesis of high-value commodities (e.g., small-molecule drugs, cosmetic ingredients, food additives, and scented compounds), and move production towards more sustainable enzyme-catalyzed processes.

The cell-free assembly of engineered metabolic pathways has led to parallel approaches in the areas of energy production, biomaterials, and even the development of artificial cells. Below we introduce some of the related efforts in these fields.

### Energy storage and generation

Cell-free enzymatic pathways have recently been used to create biobatteries with small environmental footprints and energy-storage densities superior to that of current lithium-ion devices [174]. Moreover, previous studies have demonstrated ATP generation on electrode surfaces [175, 176]. Since both the assembly of ATP synthase [113] and the synthesis of membrane proteins into tethered lipid bilayers [177] have been shown in CFS, one potential application of CFS could be rapid prototyping and construction of novel energy-generating biodevices that would be capable of producing electricity from low-value commodities (i.e., biomass or waste) [109]. One could readily imagine CFS simply powered by light [178] or electricity, which could help lower the cost of manufacturing industrially relevant biomolecules as discussed above.

### Biomaterials

As noted earlier, CFS have not only been used to screen the natural diversity of enzymes, but also to sculpt enzymatic activity. In an example of this, Bawazer et al*.* used CFS to synthesize solid-state materials [52]. A cell-free system was used to exert evolutionary selection on biomineralizing enzymes called silicateins that are capable of synthesizing silicon dioxide or titanium dioxide. DNA fragments coding for two isoforms of silicatein were digested and reassembled by DNA shuffling to create a library of chimeric enzymes. Through a clever scheme of selection, variants were then chosen for their ability to deposit silica or titanium dioxide onto microbeads in an oil-water emulsion. The success of this methodology through the use of CFS raises the exciting prospect of using green chemistry for the deposition of semi-conductor materials. This type of green deposition could also be modified such that it is guided by a CFS-compatible photolithography technique similar to that demonstrated by the Bar-Ziv group [55, 179, 180].

### Artificial cells

Artificial cells have traditionally been defined as encapsulated bioactive materials (e.g., RNA, DNA, and enzymes) within a membrane compiled to perform a designated function [134]. Incorporation of CFS into liposomes pre-dates much of the cell-free synthetic biology discussed above [181, 182] and provides a powerful platform for engineering artificial cells [37, 151, 183–185]. Artificial cells have many important applications; they can be used to link phenotype to genotype in vitro for directed evolution applications, and to spatially separate synthesis of different proteins [185]. There is also evidence indicating that confinement, a feature common to many types of artificial cells, can be used to boost protein expression yields of CFS [186]. Furthermore, artificial cells may allow for prolonged expression without relying on traditional dialysis methods that are often used to provide a continuous supply of reaction precursors. For example, early work by the Noireaux group showed that membrane-based artificial cells can be augmented with α-hemolysin pore proteins from *Staphylococcus aureus* in order to achieve selective permeability for nutrients [182, 187].

Artificial cells may also be constructed in the form of solid-state two-dimensional compartments. Silicon has been used to fabricate two-dimensional artificial cells capable of carrying out many of the features possible in cell-based systems. These features include simple metabolism, operation of gene circuits (e.g., oscillators), and even communication between compartments. Control over fabrication geometry allows for precise evaluation of the effects of diffusion gradients and can help tune protein turnover [55, 179].

Looking forward, perhaps one of the most exciting and promising applications of artificial cells is the ability to express membrane proteins efficiently. This could allow for cell-free engineering of signaling pathways [188], such as those involving G-protein-coupled receptors (GPCRs) [189, 190]. Approximately 34% of all FDA-approved drugs act on GPCR targets [191]. As such, artificial cells could become an invaluable tool in the drug discovery process. Artificial cells also have the potential to be used for in vivo therapeutics. For example, they could be designed to perform sensing, logic, or therapeutic functions. Artificial cells may be designed to accumulate at a tumor site through the enhanced permeability and retention (EPR) effect [192] or by using targeting molecules on their surface. They can also be constructed to protect therapeutic enzymes while being permeable to specific substrates and products, thus increasing active circulation time and expanding their therapeutic potential [193, 194].

## Education

Given their potential for biosafety and portability, cell-free systems offer a great platform for teaching key concepts in synthetic biology. The Cold Spring Harbor Laboratory course in synthetic biology, for example, includes modules that utilize cell-free systems [195]. In recent work led by Jim Collins and Michael Jewett, the ability of CFS to support on-demand and on-site sensing and manufacturing was further extended to bring synthetic biology capabilities to the classroom [196, 197]. Here FD-CF components were used to create kits that enable students to experience rational design of reactions, such as creating their own unique colors by mixing DNA coding for different fluorescent proteins. Other applications included the on-demand creation of fluorescent hydrogels, scents, and even sensors that could distinguish between DNA from banana, kiwi, and strawberry. Reflecting an important trend in the field of synthetic biology, this work included the testing of tools under field conditions with the help of high school students. This work sets the important groundwork for inspiring curiosity and passion in students who will drive the next generation of synthetic biology.

## The future of biotechnology with cell-free systems

The merger of cell-free systems with the vast array of genetically programmable tools is transforming the synthetic biology landscape, creating powerful in vitro platforms. These platforms have already begun to bring about de-centralization of health care through portable diagnostics and drug manufacturing. They also have great potential for the efficient, centralized production of high-value commodities. Cell-free synthetic biology approaches will take biology and biotechnology to new horizons and will surely produce many creative and unexpected outcomes. We expect the field to continue to expand and to merge with other engineered systems. One could envision programmed interactions with materials on the nano-scale and interplay with a variety of engineered enzymes. We are excited to see how CFS will bring synthetic biology closer to electronics, computation, and machine learning.

## Data Availability

Not applicable.

## References

[1] Clancy K, Voigt CA (2010). Programming cells: towards an automated ‘Genetic Compiler’. Curr Opin Biotechnol..

[2] van der Meer JR, Belkin S (2010). Where microbiology meets microengineering: design and applications of reporter bacteria. Nat Rev Microbiol..

[3] Mao N, Cubillos-Ruiz A, Cameron DE, Collins JJ (2018). Probiotic strains detect and suppress cholera in mice. Sci Transl Med.

[4] Siciliano V, DiAndreth B, Monel B, Beal J, Huh J, Clayton KL (2018). Engineering modular intracellular protein sensor-actuator devices. Nat Commun..

[5] Kotula JW, Kerns SJ, Shaket LA, Siraj L, Collins JJ, Way JC (2014). Programmable bacteria detect and record an environmental signal in the mammalian gut. Proc Natl Acad Sci U S A..

[6] Friedland AE, Lu TK, Wang X, Shi D, Church G, Collins JJ (2009). Synthetic gene networks that count. Science..

[7] Green AA, Kim J, Ma D, Silver PA, Collins JJ, Yin P (2017). Complex cellular logic computation using ribocomputing devices. Nature..

[8] Kitada Tasuku, DiAndreth Breanna, Teague Brian, Weiss Ron (2018). Programming gene and engineered-cell therapies with synthetic biology. Science.

[9] Simpson ML, Sayler GS, Fleming JT, Applegate B (2001). Whole-cell biocomputing. Trends Biotechnol..

[10] Yehl K, Lu T (2017). Scaling computation and memory in living cells. Curr Opin Biomed Eng..

[11] Anderson LA, Islam MA, Prather KLJ (2018). Synthetic biology strategies for improving microbial synthesis of “green” biopolymers. J Biol Chem..

[12] Fossati E, Ekins A, Narcross L, Zhu Y, Falgueyret J-P, Beaudoin GAW (2014). Reconstitution of a 10-gene pathway for synthesis of the plant alkaloid dihydrosanguinarine in Saccharomyces cerevisiae. Nat Commun..

[13] Smanski MJ, Zhou H, Claesen J, Shen B, Fischbach MA, Voigt CA (2016). Synthetic biology to access and expand nature’s chemical diversity. Nat Rev Microbiol..

[14] Nielsen J, Keasling JD (2016). Engineering cellular metabolism. Cell..

[15] Wagner TE, Becraft JR, Bodner K, Teague B, Zhang X, Woo A (2018). Small-molecule-based regulation of RNA-delivered circuits in mammalian cells. Nat Chem Biol..

[16] Scheller L, Strittmatter T, Fuchs D, Bojar D, Fussenegger M (2018). Generalized extracellular molecule sensor platform for programming cellular behavior. Nat Chem Biol..

[17] Cho JH, Collins JJ, Wong WW (2018). Universal chimeric antigen receptors for multiplexed and logical control of T cell responses. Cell.

[18] Lee JW, Chan CTY, Slomovic S, Collins JJ (2018). Next-generation biocontainment systems for engineered organisms. Nat Chem Biol..

[19] Jia B, Qi H, Li B-Z, Pan S, Liu D, Liu H (2017). Orthogonal ribosome biofirewall. ACS Synth Biol..

[20] Martin RW, Majewska NI, Chen CX, Albanetti TE, Jimenez RBC, Schmelzer AE (2017). Development of a CHO-based cell-free platform for synthesis of active monoclonal antibodies. ACS Synth Biol..

[21] Mikami S, Masutani M, Sonenberg N, Yokoyama S, Imataka H (2006). An efficient mammalian cell-free translation system supplemented with translation factors. Protein Expr Purif..

[22] Tran K, Gurramkonda C, Cooper MA, Pilli M, Taris JE, Selock N (2018). Cell-free production of a therapeutic protein: Expression, purification, and characterization of recombinant streptokinase using a CHO lysate. Biotechnol Bioeng..

[23] Burgenson D, Gurramkonda C, Pilli M, Ge X, Andar A, Kostov Y (2018). Rapid recombinant protein expression in cell-free extracts from human blood. Sci Rep..

[24] Ezure T, Suzuki T, Higashide S, Shintani E, Endo K, Kobayashi S-i (2006). Cell-free protein synthesis system prepared from insect cells by freeze-thawing. Biotechnol Prog..

[25] Buntru M, Vogel S, Stoff K, Spiegel H, Schillberg S (2015). A versatile coupled cell-free transcription-translation system based on tobacco BY-2 cell lysates. Biotechnol Bioeng..

[26] Harbers M (2014). Wheat germ systems for cell-free protein expression. FEBS Lett..

[27] Hodgman CE, Jewett MC (2013). Optimized extract preparation methods and reaction conditions for improved yeast cell-free protein synthesis. Biotechnol Bioeng..

[28] Yang WC, Patel KG, Wong HE, Swartz JR (2012). Simplifying and streamlining Escherichia coli-based cell-free protein synthesis. Biotechnol Prog..

[29] Kigawa T, Yabuki T, Matsuda N, Matsuda T, Nakajima R, Tanaka A (2004). Preparation of Escherichia coli cell extract for highly productive cell-free protein expression. J Struct Funct Genomics..

[30] Moore SJ, Lai H-E, Needham H, Polizzi KM, Freemont PS (2017). *Streptomyces venezuelae*TX-TL - a next generation cell-free synthetic biology tool. Biotechnol J..

[31] Moore Simon J., MacDonald James T., Wienecke Sarah, Ishwarbhai Alka, Tsipa Argyro, Aw Rochelle, Kylilis Nicolas, Bell David J., McClymont David W., Jensen Kirsten, Polizzi Karen M., Biedendieck Rebekka, Freemont Paul S. (2018). Rapid acquisition and model-based analysis of cell-free transcription–translation reactions from nonmodel bacteria. Proceedings of the National Academy of Sciences.

[32] Kelwick R, Webb AJ, MacDonald JT, Freemont PS (2016). Development of a Bacillus subtilis cell-free transcription-translation system for prototyping regulatory elements. Metab Eng..

[33] Li J, Wang H, Jewett MC (2018). Expanding the palette of Streptomyces -based cell-free protein synthesis systems with enhanced yields. Biochem Eng J..

[34] Li J, Wang H, Kwon Y-C, Jewett MC (2017). Establishing a high yielding *streptomyces*-based cell-free protein synthesis system. Biotechnol Bioeng..

[35] Failmezger J, Scholz S, Blombach B, Siemann-Herzberg M (2018). Cell-free protein synthesis from fast-growing Vibrio natriegens. Front Microbiol..

[36] Shimizu Y, Inoue A, Tomari Y, Suzuki T, Yokogawa T, Nishikawa K (2001). Cell-free translation reconstituted with purified components. Nat Biotechnol..

[37] Shin J, Noireaux V (2012). An E.coli cell-free expression toolbox: Application to synthetic gene circuits and artificial cells. ACS Synth Biol..

[38] Jewett MC, Calhoun KA, Voloshin A, Wuu JJ, Swartz JR (2008). An integrated cell-free metabolic platform for protein production and synthetic biology. Mol Syst Biol..

[39] Li J, Gu L, Aach J, Church GM (2014). Improved cell-free RNA and protein synthesis system. PLoS One..

[40] Kwon Y-C, Jewett MC (2015). High-throughput preparation methods of crude extract for robust cell-free protein synthesis. Sci Rep..

[41] Sun ZZ, Hayes CA, Shin J, Caschera F, Murray RM, Noireaux V (2013). Protocols for implementing an Escherichia coli based TX-TL cell-free expression system for synthetic biology. J Vis Exp..

[42] Caschera F, Noireaux V (2014). Synthesis of 2.3 mg/ml of protein with an all Escherichia coli cell-free transcription–translation system. Biochimie..

[43] Wiegand DJ, Lee HH, Ostrov N, Church GM. Establishing a cell-free Vibrio natriegens expression system. bioRxiv. 2018:331645. 10.1101/331645. 10.1021/acssynbio.8b00222 30160938

[44] Tuckey C, Asahara H, Zhou Y, Chong S (2014). Protein synthesis using a reconstituted cell-free system. Curr Protoc Mol Biol..

[45] Didovyk A, Tonooka T, Tsimring L, Hasty J (2017). Rapid and scalable preparation of bacterial lysates for cell-free gene expression. ACS Synth Biol..

[46] Pardee K, Green AA, Ferrante T, Cameron DE, DaleyKeyser A, Yin P (2014). Paper-based synthetic gene networks. Cell..

[47] Chan P, Thomas CJ, Sprang SR, Tall GG (2013). Molecular chaperoning function of Ric-8 is to fold nascent heterotrimeric G protein α subunits. Proc Natl Acad Sci U S A..

[48] Smith MT, Berkheimer SD, Werner CJ, Bundy BC (2014). Lyophilized Escherichia coli-based cell-free systems for robust, high-density, long-term storage. Biotechniques..

[49] Pardee K, Slomovic S, Nguyen PQ, Lee JW, Donghia N, Burrill D (2016). Portable, on-demand biomolecular manufacturing. Cell.

[50] Pardee K, Green AA, Takahashi MK, Connor DHO, Gehrke L, Collins JJ (2016). Rapid, low-cost detection of Zika virus using programmable biomolecular components. Cell..

[51] Yin G, Garces ED, Yang J, Zhang J, Tran C, Steiner AR (2012). Aglycosylated antibodies and antibody fragments produced in a scalable in vitro transcription-translation system. MAbs..

[52] Bawazer LA, Izumi M, Kolodin D, Neilson JR, Schwenzer B, Morse DE (2012). Evolutionary selection of enzymatically synthesized semiconductors from biomimetic mineralization vesicles. Proc Natl Acad Sci U S A..

[53] Sun ZZ, Yeung E, Hayes CA, Noireaux V, Murray RM (2014). Linear DNA for rapid prototyping of synthetic biological circuits in an Escherichia coli based TX-TL cell-free system. ACS Synth Biol..

[54] Takahashi MK, Chappell J, Hayes CA, Sun ZZ, Kim J, Singhal V (2015). Rapidly characterizing the fast dynamics of RNA genetic circuitry with cell-free transcription-translation (TX-TL) systems. ACS Synth Biol..

[55] Karzbrun E, Tayar AM, Noireaux V, Bar-Ziv RH (2014). Programmable on-chip DNA compartments as artificial cells. Science..

[56] Garamella J, Marshall R, Rustad M, Noireaux V (2016). The all E.coli TX-TL Toolbox 2.0: A Platform for Cell-Free Synthetic Biology. ACS Synth Biol..

[57] Niederholtmeyer H, Sun ZZ, Hori Y, Yeung E, Verpoorte A, Murray RM (2015). Rapid cell-free forward engineering of novel genetic ring oscillators. Elife..

[58] Green AA, Silver PA, Collins JJ, Yin P (2014). Toehold switches: de-novo-designed regulators of gene expression. Cell..

[59] Takahashi MK, Tan X, Dy AJ, Braff D, Akana RT, Furuta Y (2018). A low-cost paper-based synthetic biology platform for analyzing gut microbiota and host biomarkers. Nat Commun..

[60] Duyen TTM, Matsuura H, Ujiie K, Muraoka M, Harada K, Hirata K (2016). Paper-based colorimetric biosensor for antibiotics inhibiting bacterial protein synthesis. J Biosci Bioeng..

[61] Wen KY, Cameron L, Chappell J, Jensen K, Bell DJ, Kelwick R (2017). A cell-free biosensor for detecting quorum sensing molecules in *P. aeruginosa*-infected respiratory samples. ACS Synth Biol..

[62] Salehi ASM, Shakalli Tang MJ, Smith MT, Hunt JM, Law RA, Wood DW (2017). Cell-free protein synthesis approach to biosensing hTRβ-specific endocrine disruptors. Anal Chem..

[63] Salehi ASM, Yang SO, Earl CC, Shakalli Tang MJ, Porter Hunt J, Smith MT (2018). Biosensing estrogenic endocrine disruptors in human blood and urine: A RAPID cell-free protein synthesis approach. Toxicol Appl Pharmacol..

[64] Calhoun KA, Swartz JR (2005). Energizing cell-free protein synthesis with glucose metabolism. Biotechnol Bioeng..

[65] Kim T-W, Kim H-C, Oh I-S, Kim D-M (2008). A highly efficient and economical cell-free protein synthesis system using the S12 extract of Escherichia coli. Biotechnol Bioprocess Eng..

[66] Carlson ED, Gan R, Hodgman CE, Jewett MC (2012). Cell-free protein synthesis: applications come of age. Biotechnol Adv..

[67] Dudley QM, Karim AS, Jewett MC (2015). Cell-free metabolic engineering: biomanufacturing beyond the cell. Biotechnol J..

[68] Zawada JF, Yin G, Steiner AR, Yang J, Naresh A, Roy SM (2011). Microscale to manufacturing scale-up of cell-free cytokine production--a new approach for shortening protein production development timelines. Biotechnol Bioeng..

[69] Breaking free from cells; Synthetic biology. Econ. 2017 (May 6).

[70] Sullivan CJ, Pendleton ED, Sasmor HH, Hicks WL, Farnum JB, Muto M (2016). A cell-free expression and purification process for rapid production of protein biologics. Biotechnol J..

[71] Stech M, Brödel AK, Quast RB, Sachse R, Kubick S (2013). Cell-free systems: Functional modules for synthetic and chemical biology. Adv Biochem Eng Biotechnol..

[72] Brödel AK, Wüstenhagen DA, Kubick S (2015). Cell-free protein synthesis systems derived from cultured mammalian cells. Methods Mol Biol..

[73] Salehi ASM, Smith MT, Bennett AM, Williams JB, Pitt WG, Bundy BC (2016). Cell-free protein synthesis of a cytotoxic cancer therapeutic: Onconase production and a just-add-water cell-free system. Biotechnol J..

[74] Groff D, Armstrong S, Rivers PJ, Zhang J, Yang J, Green E (2014). Engineering toward a bacterial “endoplasmic reticulum” for the rapid expression of immunoglobulin proteins. MAbs..

[75] Cai Q, Hanson JA, Steiner AR, Tran C, Masikat MR, Chen R (2015). A simplified and robust protocol for immunoglobulin expression in Escherichia coli cell-free protein synthesis systems. Biotechnol Prog..

[76] Kanter G, Yang J, Voloshin A, Levy S, Swartz JR, Levy R (2007). Cell-free production of scFv fusion proteins: an efficient approach for personalized lymphoma vaccines. Blood..

[77] Stafford RL, Matsumoto ML, Yin G, Cai Q, Fung JJ, Stephenson H (2014). In vitro Fab display: a cell-free system for IgG discovery. Protein Eng Des Sel..

[78] Kawasaki T, Gouda MD, Sawasaki T, Takai K, Endo Y (2003). Efficient synthesis of a disulfide-containing protein through a batch cell-free system from wheat germ. Eur J Biochem..

[79] Stech M, Merk H, Schenk JA, Stöcklein WFM, Wüstenhagen DA, Micheel B (2013). Production of functional antibody fragments in a vesicle-based eukaryotic cell-free translation system. J Biotechnol..

[80] Xu Y, Lee J, Tran C, Heibeck TH, Wang WD, Yang J (2015). Production of bispecific antibodies in “knobs-into-holes” using a cell-free expression system. MAbs..

[81] Zimmerman ES, Heibeck TH, Gill A, Li X, Murray CJ, Madlansacay MR (2014). Production of site-specific antibody–drug conjugates using optimized non-natural amino acids in a cell-free expression system. Bioconjug Chem..

[82] Stech M, Quast RB, Sachse R, Schulze C, Wüstenhagen DA, Kubick S (2014). A continuous-exchange cell-free protein synthesis system based on extracts from cultured insect cells. PLoS One..

[83] Bulleid NJ, Bassel-Duby RS, Freedman RB, Sambrook JF, Gething MJ (1992). Cell-free synthesis of enzymically active tissue-type plasminogen activator. Protein folding determines the extent of N-linked glycosylation. Biochem J..

[84] Oh I-S, Kim D-M, Kim T-W, Park C-G, Choi C-Y (2006). Providing an oxidizing environment for the cell-free expression of disulfide-containing proteins by exhausting the reducing activity of Escherichia coli S30 extract. Biotechnol Prog..

[85] Yin G, Swartz JR (2004). Enhancing multiple disulfide bonded protein folding in a cell-free system. Biotechnol Bioeng..

[86] Palmenberg AC (1982). In vitro synthesis and assembly of picornaviral capsid intermediate structures. J Virol.

[87] Welsh JP, Lu Y, He X-S, Greenberg HB, Swartz JR (2012). Cell-free production of trimeric influenza hemagglutinin head domain proteins as vaccine antigens. Biotechnol Bioeng..

[88] Lu Y., Welsh J. P., Swartz J. R. (2013). Production and stabilization of the trimeric influenza hemagglutinin stem domain for potentially broadly protective influenza vaccines. Proceedings of the National Academy of Sciences.

[89] Zichel R, Mimran A, Keren A, Barnea A, Steinberger-Levy I, Marcus D (2010). Efficacy of a potential trivalent vaccine based on Hc fragments of botulinum toxins A, B, and E produced in a cell-free expression system. Clin Vaccine Immunol..

[90] Ng PP, Jia M, Patel KG, Brody JD, Swartz JR, Levy S (2012). A vaccine directed to B cells and produced by cell-free protein synthesis generates potent antilymphoma immunity. Proc Natl Acad Sci U S A..

[91] Bundy BC, Franciszkowicz MJ, Swartz JR (2008). Escherichia coli-based cell-free synthesis of virus-like particles. Biotechnol Bioeng..

[92] Lu Y, Chan W, Ko BY, VanLang CC, Swartz JR (2015). Assessing sequence plasticity of a virus-like nanoparticle by evolution toward a versatile scaffold for vaccines and drug delivery. Proc Natl Acad Sci U S A..

[93] Martemyanov KA, Shirokov VA, Kurnasov OV, Gudkov AT, Spirin AS (2001). Cell-free production of biologically active polypeptides: application to the synthesis of antibacterial peptide cecropin. Protein Expr Purif..

[94] Sutro Biopharma, Inc. https://www.sutrobio.com/.

[95] The Economist. Cell-free biotech will make for better products: Biotechnology. Econ. 2017.

[96] Adiga R, Al-adhami M, Andar A, Borhani S, Brown S, Burgenson D (2018). Point-of-care production of therapeutic proteins of good-manufacturing-practice quality. Nat Biomed Eng..

[97] Murphy TW, Sheng J, Naler LB, Feng X, Lu C. On-chip manufacturing of synthetic proteins for point-of-care therapeutics. Microsyst Nanoeng. 2019;25(5):13. 10.1038/s41378-019-0051-8. 10.1038/s41378-019-0051-8 PMC6431678 31057940

[98] Lu Y, Welsh JP, Chan W, Swartz JR (2013). Escherichia coli-based cell free production of flagellin and ordered flagellin display on virus-like particles. Biotechnol Bioeng..

[99] Goerke AR, Swartz JR (2008). Development of cell-free protein synthesis platforms for disulfide bonded proteins. Biotechnol Bioeng..

[100] Jiang X, Ookubo Y, Fujii I, Nakano H, Yamane T (2002). Expression of Fab fragment of catalytic antibody 6D9 in an Escherichia coli in vitro coupled transcription/translation system. FEBS Lett..

[101] Laxminarayan Ramanan, Duse Adriano, Wattal Chand, Zaidi Anita K M, Wertheim Heiman F L, Sumpradit Nithima, Vlieghe Erika, Hara Gabriel Levy, Gould Ian M, Goossens Herman, Greko Christina, So Anthony D, Bigdeli Maryam, Tomson Göran, Woodhouse Will, Ombaka Eva, Peralta Arturo Quizhpe, Qamar Farah Naz, Mir Fatima, Kariuki Sam, Bhutta Zulfiqar A, Coates Anthony, Bergstrom Richard, Wright Gerard D, Brown Eric D, Cars Otto (2013). Antibiotic resistance—the need for global solutions. The Lancet Infectious Diseases.

[102] Shin J, Jardine P, Noireaux V (2012). Genome replication, synthesis, and assembly of the bacteriophage T7 in a single cell-free reaction. ACS Synth Biol..

[103] Rustad M, Eastlund A, Jardine P, Noireaux V (2018). Cell-free TXTL synthesis of infectious bacteriophage T4 in a single test tube reaction. Synth Biol..

[104] Rustad M, Eastlund A, Marshall R, Jardine P, Noireaux V. Synthesis of infectious bacteriophages in an E. coli-based cell-free expression system. J Vis Exp. 2017;126. 10.3791/56144. 10.3791/56144 PMC5614349 28872145

[105] Potera C (2013). Phage renaissance: new hope against antibiotic resistance. Environ Health Perspect..

[106] Balogh B, Jones JB, Iriarte FB, Momol MT (2010). Phage therapy for plant disease control. Curr Pharm Biotechnol..

[107] Schlegel S, Hjelm A, Baumgarten T, Vikström D, de Gier J-W (2014). Bacterial-based membrane protein production. Biochim Biophys Acta Mol Cell Res..

[108] Schneider B, Junge F, Shirokov VA, Durst F, Schwarz D, Dötsch V (2010). Membrane protein expression in cell-free systems. Methods Mol Biol..

[109] Perez JG, Stark JC, Jewett MC (2016). Cell-free synthetic biology: Engineering beyond the cell. Cold Spring Harb Perspect Biol..

[110] Kaiser L, Graveland-Bikker J, Steuerwald D, Vanberghem M, Herlihy K, Zhang S (2008). Efficient cell-free production of olfactory receptors: Detergent optimization, structure, and ligand binding analyses. Proc Natl Acad Sci U S A..

[111] Wang X, Corin K, Baaske P, Wienken CJ, Jerabek-Willemsen M, Duhr S (2011). Peptide surfactants for cell-free production of functional G protein-coupled receptors. Proc Natl Acad Sci U S A..

[112] Fogeron M-L, Badillo A, Jirasko V, Gouttenoire J, Paul D, Lancien L (2015). Wheat germ cell-free expression: Two detergents with a low critical micelle concentration allow for production of soluble HCV membrane proteins. Protein Expr Purif..

[113] Matthies D, Haberstock S, Joos F, Dötsch V, Vonck J, Bernhard F (2011). Cell-free expression and assembly of ATP synthase. J Mol Biol..

[114] Junge F, Haberstock S, Roos C, Stefer S, Proverbio D, Dötsch V (2011). Advances in cell-free protein synthesis for the functional and structural analysis of membrane proteins. N Biotechnol..

[115] Sachse R, Dondapati SK, Fenz SF, Schmidt T, Kubick S (2014). Membrane protein synthesis in cell-free systems: From bio-mimetic systems to bio-membranes. FEBS Lett..

[116] Panganiban B, Qiao B, Jiang T, DelRe C, Obadia MM, Nguyen TD (2018). Random heteropolymers preserve protein function in foreign environments. Science..

[117] Yunker PJ, Asahara H, Hung K-C, Landry C, Arriaga LR, Akartuna I (2016). One-pot system for synthesis, assembly, and display of functional single-span membrane proteins on oil-water interfaces. Proc Natl Acad Sci U S A..

[118] Asahara H, Chong S (2010). In vitro genetic reconstruction of bacterial transcription initiation by coupled synthesis and detection of RNA polymerase holoenzyme. Nucleic Acids Res..

[119] Huppa JB, Ploegh HL (1997). In vitro translation and assembly of a complete T cell receptor-CD3 complex. J Exp Med..

[120] Matsumoto K, Tomikawa C, Toyooka T, Ochi A, Takano Y, Takayanagi N (2008). Production of yeast tRNA (m7G46) methyltransferase (Trm8–Trm82 complex) in a wheat germ cell-free translation system. J Biotechnol..

[121] Casteleijn MG, Urtti A, Sarkhel S (2013). Expression without boundaries: Cell-free protein synthesis in pharmaceutical research. Int J Pharm..

[122] Kim D-M, Swartz JR (2004). Efficient production of a bioactive, multiple disulfide-bonded protein using modified extracts of Escherichia coli. Biotechnol Bioeng..

[123] Hallam TJ, Wold E, Wahl A, Smider VV (2015). Antibody conjugates with unnatural amino acids. Mol Pharm..

[124] O’Donoghue P, Ling J, Wang Y-S, Söll D (2013). Upgrading protein synthesis for synthetic biology. Nat Chem Biol..

[125] Nguyen DP, Garcia Alai MM, Kapadnis PB, Neumann H, Chin JW (2009). Genetically encoding *N*^ϵ^ -methyl- l -lysine in recombinant histones. J Am Chem Soc..

[126] Neumann H, Hancock SM, Buning R, Routh A, Chapman L, Somers J (2009). A method for genetically installing site-specific acetylation in recombinant histones defines the effects of H3 K56 acetylation. Mol Cell..

[127] Virdee S, Kapadnis PB, Elliott T, Lang K, Madrzak J, Nguyen DP (2011). Traceless and site-specific ubiquitination of recombinant proteins. J Am Chem Soc..

[128] Alfonta L, Zhang Z, Uryu S, Loo JA, Schultz PG (2003). Site-specific incorporation of a redox-active amino acid into proteins. J Am Chem Soc..

[129] Cornish VW, Benson DR, Altenbach CA, Hideg K, Hubbell WL, Schultz PG (1994). Site-specific incorporation of biophysical probes into proteins. Proc Natl Acad Sci U S A..

[130] Oza JP, Aerni HR, Pirman NL, Barber KW, ter Haar CM, Rogulina S (2015). Robust production of recombinant phosphoproteins using cell-free protein synthesis. Nat Commun..

[131] Shozen N, Iijima I, Hohsaka T (2009). Site-specific incorporation of PEGylated amino acids into proteins using nonnatural amino acid mutagenesis. Bioorg Med Chem Lett..

[132] Patel KG, Swartz JR (2011). Surface functionalization of virus-like particles by direct conjugation using azide-alkyne click chemistry. Bioconjug Chem..

[133] Martin RW, Majewska NI, Chen CX, Albanetti TE, Jimenez RBC, Schmelzer AE (2018). Cell-free protein synthesis from genomically recoded bacteria enables multisite incorporation of noncanonical amino acids. Nat Commun..

[134] Lu Y (2017). Cell-free synthetic biology: Engineering in an open world. Synth Syst Biotechnol..

[135] Jaroentomeechai T, Stark JC, Natarajan A, Glasscock CJ, Yates LE, Hsu KJ (2018). Single-pot glycoprotein biosynthesis using a cell-free transcription-translation system enriched with glycosylation machinery. Nat Commun..

[136] Zhang Y, Ptacin JL, Fischer EC, Aerni HR, Caffaro CE, San Jose K (2017). A semi-synthetic organism that stores and retrieves increased genetic information. Nature..

[137] Hoshika S, Leal NA, Kim M-J, Kim M-S, Karalkar NB, Kim H-J (2019). Hachimoji DNA and RNA: A genetic system with eight building blocks. Science..

[138] Li H, D’Anjou M (2009). Pharmacological significance of glycosylation in therapeutic proteins. Curr Opin Biotechnol..

[139] Guarino C, DeLisa MP (2012). A prokaryote-based cell-free translation system that efficiently synthesizes glycoproteins. Glycobiology..

[140] Gibbs PEM, Zouzias DC, Freedberg IM (1985). Differential post-translational modification of human type I keratins synthesized in a rabbit reticulocyte cell-free system. Biochim Biophys Acta Gene Struct Expr..

[141] Dan S, Kang B, Duan X, Wang Y-J (2015). A cell-free system toward deciphering the post-translational modification barcodes of Oct4 in different cellular contexts. Biochem Biophys Res Commun..

[142] Kang S-H, Jun S-Y, Kim D-M (2007). Fluorescent labeling of cell-free synthesized proteins by incorporation of fluorophore-conjugated nonnatural amino acids. Anal Biochem..

[143] Pagel O, Loroch S, Sickmann A, Zahedi RP (2015). Current strategies and findings in clinically relevant post-translational modification-specific proteomics. Expert Rev Proteomics..

[144] Voigt CA, Mayo SL, Arnold FH, Wang ZG (2001). Computational method to reduce the search space for directed protein evolution. Proc Natl Acad Sci U S A..

[145] Dodevski I, Markou GC, Sarkar CA (2015). Conceptual and methodological advances in cell-free directed evolution. Curr Opin Struct Biol..

[146] Roberts RW, Szostak JW (1997). RNA-peptide fusions for the in vitro selection of peptides and proteins. Proc Natl Acad Sci U S A..

[147] Hanes J, Plückthun A (1997). In vitro selection and evolution of functional proteins by using ribosome display. Proc Natl Acad Sci U S A..

[148] Sepp A, Tawfik DS, Griffiths AD (2002). Microbead display by in vitro compartmentalisation: selection for binding using flow cytometry. FEBS Lett..

[149] Paul Siddhartha, Stang Alexander, Lennartz Klaus, Tenbusch Matthias, Überla Klaus (2012). Selection of a T7 promoter mutant with enhanced in vitro activity by a novel multi-copy bead display approach for in vitro evolution. Nucleic Acids Research.

[150] Diamante L, Gatti-Lafranconi P, Schaerli Y, Hollfelder F (2013). In vitro affinity screening of protein and peptide binders by megavalent bead surface display. Protein Eng Des Sel..

[151] Fujii S, Matsuura T, Sunami T, Kazuta Y, Yomo T (2013). In vitro evolution of α-hemolysin using a liposome display. Proc Natl Acad Sci U S A..

[152] Sumida T, Yanagawa H, Doi N (2012). In vitro selection of Fab fragments by mRNA display and gene-linking emulsion PCR. J Nucleic Acids..

[153] Adrio JL, Demain AL (2014). Microbial enzymes: tools for biotechnological processes. Biomolecules..

[154] Martin VJJ, Pitera DJ, Withers ST, Newman JD, Keasling JD (2003). Engineering a mevalonate pathway in Escherichia coli for production of terpenoids. Nat Biotechnol..

[155] Chang MCY, Eachus RA, Trieu W, Ro D-K, Keasling JD (2007). Engineering Escherichia coli for production of functionalized terpenoids using plant P450s. Nat Chem Biol..

[156] Newman JD, Marshall J, Chang M, Nowroozi F, Paradise E, Pitera D (2006). High-level production of amorpha-4,11-diene in a two-phase partitioning bioreactor of metabolically engineered Escherichia coli. Biotechnol Bioeng..

[157] Westfall PJ, Pitera DJ, Lenihan JR, Eng D, Woolard FX, Regentin R (2012). Production of amorphadiene in yeast, and its conversion to dihydroartemisinic acid, precursor to the antimalarial agent artemisinin. Proc Natl Acad Sci U S A..

[158] Galanie S, Thodey K, Trenchard IJ, Filsinger Interrante M, Smolke CD (2015). Complete biosynthesis of opioids in yeast. Science..

[159] Nakagawa A, Matsumura E, Koyanagi T, Katayama T, Kawano N, Yoshimatsu K (2016). Total biosynthesis of opiates by stepwise fermentation using engineered Escherichia coli. Nat Commun..

[160] Ajikumar PK, Xiao W-H, Tyo KEJ, Wang Y, Simeon F, Leonard E (2010). Isoprenoid pathway optimization for Taxol precursor overproduction in Escherichia coli. Science..

[161] Jagadevan S, Banerjee A, Banerjee C, Guria C, Tiwari R, Baweja M (2018). Recent developments in synthetic biology and metabolic engineering in microalgae towards biofuel production. Biotechnol Biofuels..

[162] Keasling JD (2012). Synthetic biology and the development of tools for metabolic engineering. Metab Eng..

[163] Demain AL (2000). Small bugs, big business: the economic power of the microbe. Biotechnol Adv..

[164] Jiang L, Zhao J, Lian J, Xu Z (2018). Cell-free protein synthesis enabled rapid prototyping for metabolic engineering and synthetic biology. Synth Syst Biotechnol..

[165] Chapman J, Ismail A, Dinu C (2018). Industrial applications of enzymes: Recent advances, techniques, and outlooks. Catalysts..

[166] Chen X, Zhang C, Zou R, Zhou K, Stephanopoulos G, Too HP (2013). Statistical experimental design guided optimization of a one-pot biphasic multienzyme total synthesis of amorpha-4,11-diene. PLoS One..

[167] Korman TP, Sahachartsiri B, Li D, Vinokur JM, Eisenberg D, Bowie JU (2014). A synthetic biochemistry system for the in vitro production of isoprene from glycolysis intermediates. Protein Sci..

[168] Liu T, Vora H, Khosla C (2010). Quantitative analysis and engineering of fatty acid biosynthesis in E. coli. Metab Eng..

[169] Schultheisz HL, Szymczyna BR, Scott LG, Williamson JR (2011). Enzymatic de novo pyrimidine nucleotide synthesis. J Am Chem Soc..

[170] Korman TP, Opgenorth PH, Bowie JU (2017). A synthetic biochemistry platform for cell free production of monoterpenes from glucose. Nat Commun..

[171] Sheng J, Huang L, Zhu X, Cai J, Xu Z (2014). Reconstitution of the peptidoglycan cytoplasmic precursor biosynthetic pathway in cell-free system and rapid screening of antisense oligonucleotides for Mur enzymes. Appl Microbiol Biotechnol..

[172] Zhou J, Huang L, Lian J, Sheng J, Cai J, Xu Z (2010). Reconstruction of the UDP-N-acetylglucosamine biosynthetic pathway in cell-free system. Biotechnol Lett..

[173] Karim AS, Jewett MC (2016). A cell-free framework for rapid biosynthetic pathway prototyping and enzyme discovery. Metab Eng..

[174] Zhu Z, Kin Tam T, Sun F, You C, Percival Zhang Y-H (2014). A high-energy-density sugar biobattery based on a synthetic enzymatic pathway. Nat Commun..

[175] Dobson PJ, Hill HAO, Leigh PA, Mazumdar S, Safranov AY. Adenosine triphosphate synthesis using an electrochemically-driven proton pump. J Chem Soc Chem Commun. 1994;0(7):807. doi: 10.1039/c39940000807

[176] Gutiérrez-Sanz Ó, Natale P, Márquez I, Marques MC, Zacarias S, Pita M (2016). H_2_-fueled ATP synthesis on an electrode: Mimicking cellular respiration. Angew Chemie Int Ed..

[177] Zieleniecki JL, Nagarajan Y, Waters S, Rongala J, Thompson V, Hrmova M (2016). Cell-free synthesis of a functional membrane transporter into a tethered bilayer lipid membrane. Langmuir..

[178] Berhanu S, Ueda T, Kuruma Y (2019). Artificial photosynthetic cell producing energy for protein synthesis. Nat Commun..

[179] Buxboim A, Bar-Dagan M, Frydman V, Zbaida D, Morpurgo M, Bar-Ziv R (2007). A single-step photolithographic interface for cell-free gene expression and active biochips. Small..

[180] Bar M, Bar-Ziv RH (2009). Spatially resolved DNA brushes on a chip: gene activation by enzymatic cascade. Nano Lett..

[181] Ishikawa K, Sato K, Shima Y, Urabe I, Yomo T (2004). Expression of a cascading genetic network within liposomes. FEBS Lett..

[182] Noireaux V, Libchaber A (2004). A vesicle bioreactor as a step toward an artificial cell assembly. Proc Natl Acad Sci U S A..

[183] Kuruma Y, Stano P, Ueda T, Luisi PL (2009). A synthetic biology approach to the construction of membrane proteins in semi-synthetic minimal cells. Biochim Biophys Acta Biomembr..

[184] Wu F, Tan C (2014). The engineering of artificial cellular nanosystems using synthetic biology approaches. Wiley Interdiscip Rev Nanomed Nanobiotechnol..

[185] Elani Y, Law RV, Ces O (2015). Protein synthesis in artificial cells: using compartmentalisation for spatial organisation in vesicle bioreactors. Phys Chem Chem Phys..

[186] Sakamoto R, Noireaux V, Maeda YT (2018). Anomalous scaling of gene expression in confined cell-free reactions. Sci Rep..

[187] Noireaux Vincent, Bar-Ziv Roy, Godefroy Jeremy, Salman Hanna, Libchaber Albert (2005). Toward an artificial cell based on gene expression in vesicles. Physical Biology.

[188] Ho KKY, Murray VL, Liu AP (2015). Engineering artificial cells by combining HeLa-based cell-free expression and ultrathin double emulsion template. Methods Cell Biol..

[189] Segers K, Masure S (2015). Cell-free expression of G protein-coupled receptors. Curr Protoc Protein Sci..

[190] Sonnabend A, Spahn V, Stech M, Zemella A, Stein C, Kubick S (2017). Production of G protein-coupled receptors in an insect-based cell-free system. Biotechnol Bioeng..

[191] Hauser AS, Attwood MM, Rask-Andersen M, Schiöth HB, Gloriam DE (2017). Trends in GPCR drug discovery: new agents, targets and indications. Nat Rev Drug Discov..

[192] Usmani A, Mishra A, Ahmad M (2018). Nanomedicines: a theranostic approach for hepatocellular carcinoma. Artif Cells Nanomed Biotechnol..

[193] Chang TMM (1964). Semipermeable microcapsules. Science..

[194] Xu C, Hu S, Chen X (2016). Artificial cells: from basic science to applications. Mater Today (Kidlington)..

[195] Cold Spring Harbor Laboratory Course in Synthetic Biology [Internet] (https://meetings.cshl.edu/courses.aspx?course=C-SYNBIO&year=19).

[196] Huang A, Nguyen PQ, Stark JC, Takahashi MK, Donghia N, Ferrante T (2018). BioBits^TM^ Explorer: A modular synthetic biology education kit. Sci Adv.

[197] Stark JC, Huang A, Nguyen PQ, Dubner RS, Hsu KJ, Ferrante TC (2018). BioBits^TM^ Bright: A fluorescent synthetic biology education kit. Sci Adv.

